# Patient Characteristics Influence Activated Signal Transducer and Activator of Transcription 3 (STAT3) Levels in Primary Breast Cancer—Impact on Prognosis

**DOI:** 10.3389/fonc.2020.01278

**Published:** 2020-07-29

**Authors:** Linn Nilsson, Emma Sandén, Somayeh Khazaei, Helga Tryggvadottir, Björn Nodin, Karin Jirström, Signe Borgquist, Karolin Isaksson, Helena Jernström

**Affiliations:** ^1^Department of Clinical Sciences in Lund, Oncology, Lund University and Skåne University Hospital, Lund, Sweden; ^2^Department of Medical Physics and Engineering, Växjö Central Hospital, Växjö, Sweden; ^3^Department of Research and Development, Region Kronoberg, Växjö, Sweden; ^4^Department of Oncology, Aarhus University and Aarhus University Hospital, Aarhus, Denmark; ^5^Department of Clinical Sciences in Lund, Surgery, Lund University, Lund, Sweden; ^6^Department of Surgery, Central Hospital Kristianstad, Kristianstad, Sweden

**Keywords:** signal transducer and activator of transcription 3 (STAT3), breast cancer, prognosis, immunohistochemistry, tamoxifen, lifestyle

## Abstract

**Background:** Activated signal transducer and activator of transcription 3 (pSTAT3) is often present in breast cancer, but its prognostic impact is still unclear. We investigated how breast tumor-specific pSTAT3^Y705^ levels are associated with patient and tumor characteristics and risk of recurrence.

**Materials and Methods:** Primary breast cancer patients without preoperative treatment were included preoperatively. The patients were treated in Lund, Sweden, in 2002–2012 and followed until 2016. Levels of pSTAT3^Y705^ were evaluated in 867 tumors using tissue microarrays with immunohistochemistry and categorized according to the H-score as negative (0–9; 24.2%), intermediate (10–150; 69.9%), and high (160–300; 5.9%).

**Results:** Patients were followed for up to 13 years, and 137 recurrences (88 distant) were recorded. Higher pSTAT3^Y705^ levels were associated with patient characteristics including younger age, any alcohol consumption, higher age at first child birth, and smaller body size, as well as tumor characteristics including smaller tumor size, lower histological grade, lymph node negativity, progesterone receptor positivity, and HER2 negativity (all *P*_trends_ ≤ 0.04). Higher pSTAT3^Y705^ levels were associated with lower risk of early recurrences (LogRank *P*_trend_ = 0.10; 5-year LogRank *P*_trend_ = 0.004) and distant metastases (LogRank *P*_trend_ = 0.045; 5-year LogRank *P*_trend_ = 0.0007), but this was not significant in the multivariable models. There was significant effect modification between tamoxifen treatment and pSTAT3^Y705^ negativity on the recurrence risk in chemonaïve patients with estrogen receptor positive tumors [adjusted hazard ratio (HR) 0.38; *P*_interaction_ = 0.046].

**Conclusion:** Higher pSTAT3^Y705^ levels were associated with several patient and tumor characteristics that are mainly associated with good prognosis and a tendency toward lower risk for early recurrences. In the future, these results may help guide the selection of patients for trials with drugs targeting the STAT3 pathway.

## Introduction

Breast cancer is a heterogeneous disease with treatments comprising different modalities to target specific tumor characteristics. In order to improve the prognosis, new adjuvant treatments in addition to surgery and treatment-predictive markers are warranted. The signal transducer and activator of transcription 3 (STAT3) is one of seven members of the STAT family, and several STATs are known to be involved in different stages of normal breast development. STAT3 is located on chromosome 17q21 (1) and regulates the expression of genes associated with cell death, proliferation, angiogenesis, and inflammation. Furthermore, it specifically mediates mammary gland regression and tissue remodeling during involution (2).

Activation occurs by phosphorylation at tyrosine 705 or serine 727 (pSTAT3), most commonly by cytokine interleukin-6 (IL-6) through the janus kinas 2 (JAK2)/STAT3 pathway. However, STAT3 can also be activated by other cytokines and growth factors such as epidermal growth factor (EGF) (3) and leptin (4). STAT3 regulates many genes associated with proliferation, invasiveness and vascularization, for example the genes encoding cyclin D1, matrix metalloproteinases (MMP), vascular endothelial growth factor (VEGF), and PD-L1 (1, 5, 6). As an acute phase protein, the activation of STAT3 is transient under normal conditions, but in tumor cells, there is a constitutive activation (7). STAT3 is also known to impact the tumor immune response (6) and cancer stem cells (4).

Elevated levels of STAT3 or pSTAT3 have been demonstrated in a range of solid cancer forms and are mainly associated with poor prognosis (8). In breast cancer, the prognostic association is less clear (9), and levels of pSTAT3 in hyperplasia and tumor tissue compared with normal tissue show inconclusive results (5, 10–12). The presence of pSTAT3 has been shown irrespective of hormone receptor status in breast cancer (10, 13). However, STAT3 has mainly been found in basal-like breast cancer cells, CD44+ CD24–, in which the secretion of IL-6 increases the level of pSTAT3 through an autocrine growth regulator loop (14).

The pSTAT3 pathway is suppressed by either “protein inhibitor of activated STAT3” (PIAS3), which is activated by STAT3 itself, or “suppression of cytokine signaling 3” (SOCS3). There are also natural inhibitors such as caffeic acid and curcumin (1). Activation of STAT3 has been suggested to be involved in resistance to chemotherapy (4), tamoxifen (15), radiotherapy (16), and trastuzumab treatment (17). Moreover, one study showed that pSTAT3 levels were elevated after neoadjuvant chemotherapy (18). Given the central role in tumorigenesis and treatment resistance, STAT3 is a tentative prognostic marker and may also serve as a molecular target for therapy. In metastatic breast cancer, one clinical trial inhibiting STAT3 via a tyrosine kinase inhibitor targeting JAK1/2 (ruxolitinib) has been conducted (19), and other clinical trials are under way (NCT03195699, NCT02876302).

STAT3 plays a central role in tumorigenesis, but to the best of the authors' knowledge, no studies have investigated the association between levels of pSTAT3 and lifestyle factors in breast cancer. Furthermore, little is known about how pSTAT3 impacts prognosis in different treatment groups of primary breast cancer. Therefore, the aim of this study was to investigate how the level of pSTAT3 is linked to patient and tumor characteristics and clinical outcomes in primary breast cancer patients following different treatments in a Swedish cohort of breast cancer patients.

## Materials and Methods

### Study Population

The BC-blood study is an ongoing prospective population-based study with the aim of elucidating factors that may have prognostic or predictive values for breast cancer patients. Women diagnosed with a first breast cancer were enrolled at Skåne University Hospital in Lund, Sweden, between October 2002 and June 2012. Patients with a previous cancer diagnosis within the last 10 years were not included. Ethical approval was obtained from the Ethics Committee of Lund University (Dnr75–02, Dnr37–08, Dnr658–09, Dnr58–12, Dnr379–12, Dnr227–13, Dnr277–15, and Dnr458–15), and written informed consent was obtained from all participants.

**Figure 1:**
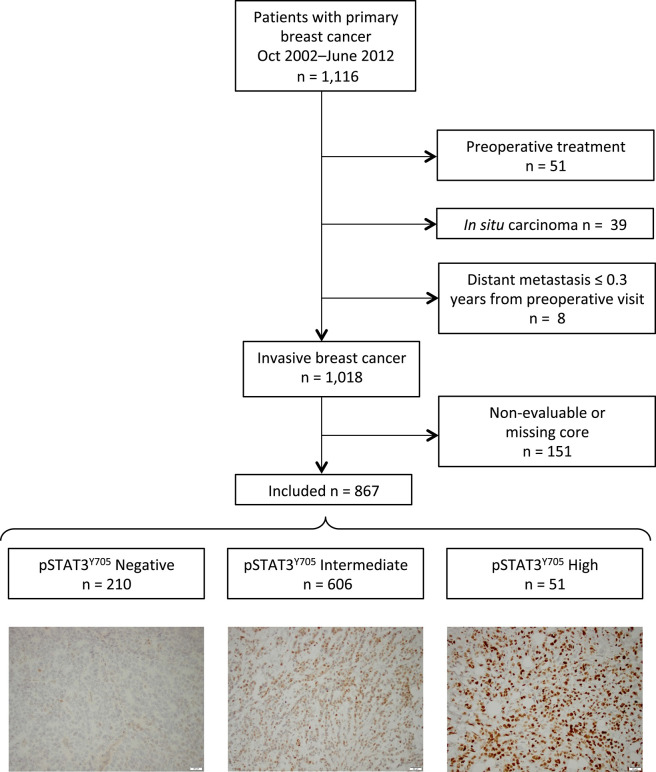
Flow chart of included and excluded patients and representative images of pSTAT3 staining intensities.

The current study is based on 1,116 patients, and flowcharts of included and excluded patients are shown in Figure 1. The final study population included in the analyses of pSTAT3^Y705^ consisted of 867 patients with primary invasive breast cancer who had not received preoperative treatment. All included patients completed preoperative and post-operative questionnaires including questions on reproductive factors, such as age at menarche, number of children, maternal age at the birth of the first and last child, use of oral contraceptives (OC) and menopausal hormone therapy (MHT).

Current or ever users of OC or MHT were considered ever users regardless of the duration of use. Patients were classified as MHT users if they had indicated hormone use for menopausal symptoms. However, progestin-containing intrauterine devices were not considered as MHT (20). Furthermore, information on preoperative smoking habits (current or occasional smoker vs. non-smoker), coffee consumption (21), and alcohol use was recorded according to the AUDIT (alcohol use disorders identification test) questionnaire (22).

Anthropometric measures were obtained preoperatively by trained research nurses. The body mass index (BMI) was calculated as the weight in kilograms divided by the square of the person's height in meters. The cut-off for overweight (BMI ≥25 kg/m^2^) was set according to the world health organization (WHO) classification (23). The waist-to-hip ratio (WHR) was defined as the ratio of the circumference of the waist to the widest part of the hip, and the cut-off for central obesity was set as >0.85 (24).

Breast volume was measured using plastic cups developed for breast measurements prior to breast reduction and reconstruction (25). A sum of the left and right breast volumes ≥850 ml was used to distinguish between small and large breast volumes as described previously in this cohort (26). Mammography-detected tumors in patients aged 45–74 years were considered screening-detected tumors since this age group was regularly invited for mammographic screening during the entire period of inclusion.

Information on tumor characteristics was obtained from pathology reports and included tumor size, axillary lymph node involvement, histological grade and type, and hormone status; estrogen (ER) and progesterone (PR). Tumors were classified as ductal, lobular, or other and mixed (tumors with a mixed ductal and lobular type were included in the last category). Tumors with >10% ER^+^ or PR^+^ nuclei staining were considered hormone receptor positive (27) according to recommendations in clinical practice in Sweden.

Human epidermal growth factor receptor 2 (HER2) status was incorporated into clinical routine in November 2005 and was available for the majority of tumors after that date. Clinical HER2 status was supplemented with HER2 status obtained by retrospective HER2 analysis with gene protein assay using tissue microarrays (TMA) for 269 patients. The agreement in HER2 status between the pathological assessment using whole slides and TMA was 97.7% (28).

Information on adjuvant breast cancer treatment was recorded until the time of the first breast cancer event, death, or the last follow-up and was collected from patient charts and questionnaires. Most patients received more than one treatment modality after surgery. Any chemotherapy was administered prior to radiation therapy or endocrine treatment. Information on breast cancer events during follow-up was acquired from patient charts and the Regional Tumor Registry, and the date of death was acquired from clinical charts or the Swedish Population Registry.

### Tissue Microarray and Immunohistochemistry of pSTAT3

Tissue microarrays (TMA) of breast cancer were constructed using a semi-automated tissue array device (Beecher Instruments, Sun Prairie, WI, USA). Duplicate 1-mm cores were sampled from representative tumor regions of formalin-fixed paraffin-embedded tissues. All staining of the TMAs was performed using the same protocol. Sections of 4 μm TMA were deparaffinized, pretreated using an automatic PT-link system (DAKO, Glostrup, Denmark), and stained using a pSTAT3 antibody (rabbit anti-STAT3 phospho Y705, ab76315, diluted 1:100 for 30 min at pH9, Abcam, Cambridge, UK) and EnVision FLEX high-pH kit (10 min development time) in an Autostainer Plus according to the manufacturer's instructions (DAKO, Glostrup, Denmark). The tumor-specific pSTAT3^Y705^ level was evaluated by two independent observers (ES and LN), who were blinded to the information regarding clinical data and outcomes. A senior pathologist (KJ) was available for consultation. Duplicate cores were evaluated jointly across both cores. In cases that showed discrepancy between the two observers, a reevaluation was performed, and consensus was reached. Tumor-adjacent normal ducts were negative for pSTAT3 and placenta (known to be rapidly proliferating) was positive.

Nuclear staining of pSTAT3^Y705^ in invasive tumor cells was assessed as the fraction and intensity of stained nuclei. Cores with low numbers of invasive tumor cells (<50 cells), cores with staining artifacts, and missing cores were annotated as non-evaluable. Since the staining was highly heterogenic, a Histo-score (H-score) (10) was calculated for each tumor, where the fraction in steps of 10 stained cells (0–100%) was multiplied by its staining intensity (0–3). This resulted in a range of H-scores between 0 and 300. The study adhered to the reporting recommendations for tumor marker prognostic study (REMARK) (29) criteria.

### Statistical Methods

All statistical analyses were performed using SPSS software version 24 (IBM Corp, Armonk, NY, USA). Patient and tumor characteristics are presented with descriptive statistics as either continuous (median and interquartile range) or categorical (number or percent). Discrepancies between evaluated and non-evaluated patients regarding patient and tumor characteristics were analyzed using the Mann-Whitney *U*-test (continuous) or Chi-square test (categorical).

Variables were dichotomized as follows: age at inclusion (≥50 years), BMI (≥25 kg/m^2^), WHR (>0.85), breast volume (≥850 mL), ever use of OC (yes), parous (yes), ever MHT (yes), current smoker (yes), alcohol abstainer (yes), coffee consumption (≥2 cups/day), tumor size (≥21 mm or skin or muscular involvement), any axillary lymph node involvement (yes), histological grade III (yes), ER^+^, PR^+^, or HER2^+^ (yes), screening detected (yes), and ever adjuvant chemo- or radiotherapy (yes).

The level of pSTAT3^Y705^ was divided into three categories: negative (0–9), intermediate (10–150), and high (160–300). In the case of bilateral tumors (*n* = 18), pSTAT3^Y705^ from both tumors was available for eight tumors, and the level of pSTAT3^Y705^ differed in three of these tumors. For these three tumors, the tumor from the side with the highest pSTAT3^Y705^ level was used, and all tumor characteristics were taken from the corresponding side.

The Jonckheere trend test (continuous variables) or Chi-square test for linear trends (categorical variables) was used for the analysis between different levels of pSTAT3^Y705^ in relation to patient and tumor characteristics. Since phosphorylated immunohistochemical markers can be sensitive to long-term storage in paraffin (30), an analysis of the time between surgery and staining in full years (31) was performed using Spearman correlation.

Patients were followed until June 30, 2016, and Kaplan-Meier curves and LogRank tests were used for univariable survival analyses for three different end points: any breast cancer event, distant metastases, and death. Breast cancer-free interval was defined as the time from inclusion until any breast cancer event, with follow-up time censored at death or last follow-up. The distant metastasis-free interval was defined as the time from inclusion until the first distant metastasis and censored at death or last follow-up.

Overall survival was defined as the time from inclusion until death or the last follow-up. Cox regressions for crude and adjusted pSTAT3^Y705^ in different models were used to determine hazard ratios (HR) and presented with 95% confidence intervals (CIs). Model 1 was adjusted for the time between surgery and staining. Model 2 was adjusted for the time between surgery and staining, age (≥50 years), tumor characteristics, and BMI (≥25 kg/m^2^). Model 3 includes model 2 and further adjustments for adjuvant treatments (chemotherapy, radiotherapy, endocrine therapy, and trastuzumab).

As the Kaplan-Meier curves were not proportional over time, the LogRank was calculated for the 5- and 10-year follow-up. Hazard ratios were calculated for the 5-year follow-up and the entire follow-up period. To analyze the effect modifications, multiplicative interaction variables were created between the pSTAT3^Y705^ categories “negative” or “high” (with intermediate as a reference) and age (≥50 years), body constitution, BMI (≥ 25kg/m^2^), WHR (>0.85), breast volume (≥850 mL), tumor characteristics, tumor size (≥21 mm or skin or muscular involvement), any axillary lymph node involvement, histological grade (III), ER^+^- PR^+^ and HER2 status (positive), triple-negative breast cancer, and treatments (chemotherapy, aromatase inhibitors, tamoxifen, radiation therapy, and trastuzumab).

Power calculations were performed using PS Power Sample Size Calculation Program, version 3.1.2. For the power calculation, we assumed that with 860 patients with 25% negative for pSTAT3^Y705^, a 10-year accrual interval, an additional 4-year follow-up, and a median follow-up of 5 years, we would be able to detect true HRs of 0.779 or 1.306 with 80% power at a significance level of 0.05. All *P*-values were two-tailed, and *P* < 0.05 were considered statistically significant. Since this was an exploratory study, nominal *P*-values are presented without adjustments for multiple testing (32).

## Results

### Activated STAT3 in Relation to Patient Characteristics

Patient characteristics and associations with pSTAT3^Y705^ are shown in Table 1. The patients' ages ranged from 24 to 99 years with a median of 61 years. For the 867 patients with tumors available for pSTAT3^Y705^ scoring, 51 (5.9%) patients had a high level, 606 (69.9%) had an intermediate level, and 210 (24.2%) were negative regarding pSTAT3^Y705^. A correlation was found between longer time since surgery and a weaker pSTAT3^Y705^ H-score (*R*_s_ = −0.132; *P* = 0.00009), hence adjustment for time between surgery and staining was performed.

**Table 1:** Patient characteristics at inclusion in relation to pSTAT3^Y705^ levels.

			**pSTAT3**^****Y705****^ **level**			
	**All**	**Missing**	**Negative**	**Intermediate**	**High**	**Non-evaluable**
	***n* = 867**		***n* = 210 (24.2%)**	***n* = 606 (69.9%)**	***n* = 51 (5.9%)**	***n* = 151**
	**Median (IQR) or %**		**Median (IQR) or %**	**Median (IQR) or %**	**Median (IQR) or %**	**Median (IQR) or %**
Age at inclusion, years	61.1 (52.2–68.1)	0	61.6 (54.0–69.4)	61.0 (51.7–68.1)	59.0 (51.7–67.4)	61.2 (51.9–68.1)
Age of 50 years or older at inclusion	698 (80.5)	0	179 (85.2)	476 (78.5)	43 (84.3)	118 (78.1)
Age at menarche, years	13 (12–14)	6	13 (12–14)	13 (12–14)	13 (12–14)	13 (13–14)
Height, meters	1.66 (1.62–1.70)	23	1.65 (1.62–1.70)	1.66 (1.62–1.70)	1.65 (1.60–1.69)	1.65 (1.62–1.68)
Weight, kgs	69.0 (62.0–78.0)	23	72.0 (64.0–82.8)	69 (62.0–76.5)	62.3 (54.7–73.0)	69.4 (61.2–78.9)
BMI	25.1 (22.5–28.3)	25	26.2 (23.2–29.8)	24.8 (22.4–27.9)	24.0 (20.6–26.2)	25.1 (22.3–28.5)
BMI ≥25 kg/m^2^	428 (50.8)	25	126 (61.5)	284 (48.3)	18 (36.7)	75 (50.7)
Waist to hip ratio	0.85 (0.81–0.90)	29	0.87 (0.82–0.92)	0.85 (0.80–0.90)	0.84 (0.79–0.90)	0.87 (0.81–0.90)
Waist to hip ratio >0.85	436 (52.0)	29	120 (58.8)	297 (50.8)	19 (38.8)	83 (58.5)
Total breast volume, mL^*^	1,000 (650–1,500)	127	1,150 (700–1,800)	950 (650–1,450)	800 (362–1,000)	1,000 (669–1,000)
Breast volume ≥850 mL^*^	419 (56.6)	127	124 (67.8)	275 (53.6)	20 (45.5)	73 (61.9)
Ever use of OC	613 (70.8)	1	153 (73.2)	425 (70.1)	35 (68.6)	109 (72.2)
Parous	760 (87.8)	1	180 (85.7)	538 (88.9)	42 (82.4)	136 (90.1)
Parity	2 (1–3)	1	2 (1–3)	2 (1–3)	2 (1–3)	2 (1–2)
Age of mother at first child	25 (22–28)	113	24 (20–27)	25 (22–28)	27 (23–29)	25 (22–28)
Age of mother at last child	30 (27–34)	110	30 (26–34)	30 (27–33)	31 (28–35)	29 (26–34)
Ever treatment for menopausal symptoms	379 (43.9)	3	95 (45.7)	258 (42.6)	26 (51.0)	68 (45.0)
Current smoker	169 (19.5)	2	49 (23.3)	111 (18.4)	9 (17.6)	37 (24.5)
Alcohol abstainer	94 (10.9)	2	28 (13.3)	65 (10.8)	1 (2.0)	12 (8.0)
Coffee consumption, ≥2 cups/day	698 (80.9)	4	170 (81.0)	489 (81.2)	39 (76.5)	126 (83.4)

* *In patients without previous breast surgeries*.

Regarding patient characteristics, there was a trend between increasing pSTAT3^Y705^ levels and younger age at inclusion (*P*_trend_ = 0.026), lower weight (*P*_trend_ < 0.0001), lower BMI (*P*_trend_ = 0.0001), lower WHR (*P*_trend_ = 0.0003), smaller breast volume (*P*_trend_ < 0.0001), older age at first childbirth (*P*_trend_ = 0.004), and lower frequency of alcohol abstention (*P*_trend_ = 0.041). The remaining patient characteristics were not statistically associated with the level of pSTAT3^Y705^. The only difference regarding patients with evaluated and non-evaluated tumors was the number of children. All *P*-values were adjusted for time between surgery and staining and remained essentially the same in the unadjusted model except for age at inclusion, which was non-significant in the unadjusted model.

### Activated STAT3 in Relation to Tumor Characteristics

Higher pSTAT3^Y705^ levels were associated with smaller tumors (*P*_trend_ = 0.018), fewer involved lymph nodes (*P*_trend_ = 0.016), lower histological grade (*P*_trend_ < 0.0001), PR^+^ (*P*_trend_ = 0.001), and HER2 negativity (*P*_trend_ = 0.011). Higher pSTAT3^Y705^ levels were associated with a higher frequency of the lobular type (*P*_trend_ = 0.001) and a lower frequency of the ductal type (*P*_trend_ = 0.038). All *P*-values were adjusted for time between surgery and staining, and there was no statistical association between the pSTAT3^Y705^ levels and screening-detection or adjuvant treatments (see Table 2). Tumors not evaluated for pSTAT3^Y705^ due to missing tissue had more favorable tumor characteristics and were less often of the lobular type, and patients with such tumors were more likely to have been treated with aromatase inhibitors than patients with evaluable tumors.

**Table 2:** Tumor characteristics at inclusion in relation to pSTAT3^Y705^ levels, tumor characteristics are presented for the same side as the highest pSTAT3^Y705^ level in case of bilateral tumors.

		**pSTAT3**^****Y705****^ **level**			**Non-evaluable**
	**All**	**Negative**	**Intermediate**	**High**	
	***n* = 867**	***n* = 210 (24.2%)**	***n* = 606 (69.9%)**	***n* = 51 (5.9%)**	***n* = 151**
	**Number (%)**	**Number (%)**	**Number (%)**	**Number (%)**	**Number (%)**
**Invasive tumor size**					
1–20 mm	619 (71.4)	135 (64.3)	442 (72.9)	42 (82.4)	124 (82.1)
21–50 mm	233 (26.9)	70 (33.3)	157 (25.9)	6 (11.8)	26 (17.2)
≥51 mm	13 (1.5)	5 (2.4)	7 (1.2)	1 (2.0)	1 (0.7)
Skin or muscular involvement	2 (0.2)	0	0	2 (3.9)	0
Missing	0	0	0	0	0
≥21 mm or skin or muscular involvement	248 (28.6)	75 (35.7)	164 (27.1)	9 (17.6)	27 (17.9)
**Axillary nodal involvement**					
0	526 (60.8)	114 (54.8)	378 (62.4)	34 (66.7)	103 (68.2)
1–3	266 (30.8)	69 (33.2)	183 (30.2)	14 (27.5)	36 (23.8)
≥4	73 (8.4)	25 (12.0)	45 (7.4)	3 (5.9)	12 (7.9)
Missing	2	2	0	0	0
Any axillary lymph node involvement	339 (39.2)	94 (45.2)	228 (37.6)	17 (33.3)	48 (31.8)
**Histological grade**					
I	204 (23.5)	30 (14.3)	159 (26.2)	15 (29.4)	54 (36.0)
II	434 (50.1)	109 (51.9)	294 (48.5)	31 (60.8)	68 (45.3)
III	229 (26.4)	71 (33.8)	153 (25.2)	5 (9.8)	28 (18.7)
Missing	0	0	0	0	1
Histologic grade III	229 (26.4)	71 (33.8)	153 (25.2)	5 (9.8)	28 (18.7)
**Histological type**					
Ductal mainly	713 (82.2)	178 (84.8)	499 (82.3)	36 (70.6)	111 (73.5)
Lobular mainly	96 (11.1)	15 (7.1)	68 (11.2)	13 (25.5)	21 (13.9)
Other or mixed	58 (6.7)	17 (8.1)	39 (6.4)	2 (3.9)	19 (12.6)
Missing	0	0	0	0	0
**Hormone receptor status**					
ER+	762 (88.0)	181 (86.6)	534 (88.1)	47 (92.2)	132 (87.4)
Missing	1	1	0	0	0
PR+	610 (70.4)	127 (60.8)	443 (73.1)	40 (78.4)	111 (73.5)
Missing	1	1	0	0	0
**HER2 status**					
HER2+	89 (10.6)	32 (15.7)	53 (9.0)	4 (8.3)	16 (14.5)
Not analyzed	25	6	6	3	41
**Triple negative**					
Triple negative	68 (7.9)	15 (7.2)	51 (8.4)	2 (3.9)	6 (4.2)
Missing	1	1	0	0	7
**Screening 45–74 years**					
Screening detected	474 (64.9)	111 (62.7)	337 (66.3)	26 (57.8)	95 (73.6)
Missing	0	0	0	0	0
**Treatments by last follow-up prior to any event**					
Chemotherapy	225 (26.0)	60 (28.6)	154 (25.4)	11 (21.6)	34 (22.5)
Radiotherapy	551 (63.6)	136 (64.8)	386 (63.7)	29 (56.9)	93 (61.6)
**ER+** **tumors only**					
Tamoxifen	478 (62.9)	118 (65.2)	333 (62.5)	27 (58.7)	73 (55.3)
Aromatase inhibitors	320 (42.0)	83 (45.9)	219 (41.0)	18 (39.1)	43 (32.6)
**HER2 amplified only**					
Trastuzumab^*^	56 (62.9)	20 (62.5)	35 (66.0)	1 (25.0)	11 (68.3)
**Type of event**					
Any breast cancer event	137 (15.8)	40 (19.0)	95 (15.7)	2 (3.9)	19 (12.6)
Distant metastasis	88 (10.1)	30 (14.3)	56 (9.2)	2 (3.9)	11 (7.3)
Death	113 (13.0)	36 (17.1)	72 (11.9)	5 (9.8)	15 (9.9)

*ER, estrogen receptor; PR, progesterone receptor; HER2, human epidermal growth factor receptor*.

* *Three additional patients with intermediate pSTAT3 level had a HER2+ tumor on the contralateral side and also received trastuzumab*.

### Activated STAT3 and Prognosis

Patients were followed for up to 13 years, and for the 681 patients still at risk, the median follow-up time was 5.4 years (interquartile range 4.2–9.1). During follow-up, there was a total of 137 recurrences, of which 88 were distant metastasis (Table 2). For 75 of these 88 patients, the distant metastasis was the first event. Of the 113 patients who died during the study period, 64 (56.6%) had a previous recurrence, and 49 (43.4%) patients died without a previous recurrence.

**Figure 2:**
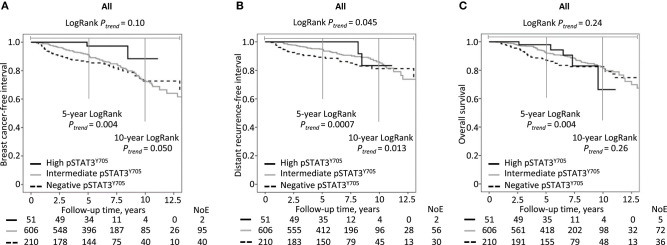
Kaplan-Meier estimates of **(A)** breast cancer-free interval, **(B)** distant metastasis-free interval and **(C)** overall survival in relation to pSTAT3^Y705^ level in all patients.

Higher pSTAT3^Y705^ levels were associated with somewhat longer breast cancer-free intervals (LogRank *P*_trend_ = 0.10) and distant metastasis-free intervals (LogRank *P*_trend_ = 0.045), but not overall survival (LogRank *P*_trend_ = 0.24; see Figure 2). The impact of pSTAT3^Y705^ levels on clinical outcomes was mainly pronounced within the first 5 years. Table 3 shows crude and adjusted HRs for pSTAT3^Y705^ in relation to clinical outcomes for the entire follow-up and the 5-year follow-up. The associations between pSTAT3^Y705^ levels and clinical outcomes became weaker after adjustments for patient and tumor characteristics and remained essentially the same after further adjustments for treatments.

**Table 3:** Multivariable models of crude and adjusted HR (95% CIs) for breast cancer recurrence, distant metastasis, and overall survival for the entire follow-up and 5-year follow-up in relation to pSTAT3^Y705^ level.

**BREAST CANCER RECURRENCE**																		
							**Model 1**				**Model 2**				**Model 3**			
	**Total**	**Events**	**Crude**				**Adjusted**				**Adjusted**				**Adjusted**			
Tumor pSTAT3^Y705^ status	***n***	***n***	**HR**	**(95% CI)**		***P*****-value**	**HR**	**(95% CI)**		***P*****-value**	**HR**	**(95% CI)**		***P*****-value**	**HR**	**(95% CI)**		***P*****-value**
Negative	210	40	Ref				Ref				Ref				Ref			
Intermediate	606	95	0.89	0.61	1.28	0.53	0.91	0.63	1.32	0.62	1.13	0.76	1.67	0.54	1.13	0.76	1.68	0.54
High	51	2	0.24	0.06	1.00	0.051	0.26	0.06	1.07	0.061	0.41	0.10	1.74	0.23	0.39	0.09	1.62	0.19
		***P***_**trend**_	0.10				0.14				0.89				0.84			
Tumor pSTAT3^Y705^ status	***n***	***n***	**HR 5-year**				**HR 5-year**				**HR 5-year**				**HR 5-year**			
Negative	210	27	Ref				Ref				Ref				Ref			
Intermediate	606	47	0.58	0.36	0.94	0.026	0.64	0.40	1.02	0.063	0.82	0.50	1.35	0.43	0.80	0.48	1.32	0.38
High	51	1	0.14	0.02	1.07	0.058	0.17	0.02	1.25	0.082	0.29	0.04	2.18	0.23	0.28	0.04	2.07	0.21
		***P***_**trend**_	0.004				0.014				0.22				0.18			
**DISTANT METASTASIS**																		
							**Model 1**				**Model 2**				**Model 3**			
	**Total**	**Events**	**Crude**				**Adjusted**				**Adjusted**				**Adjusted**			
Tumor pSTAT3^Y705^ status	**n**	**n**	**HR**	**(95% CI)**		***P*****-value**	**HR**	**(95% CI)**		***P*****-value**	**HR**	**(95% CI)**		***P*****-value**	**HR**	**(95% CI)**		***P*****-value**
Negative	210	30	Ref				Ref				Ref				Ref			
Intermediate	606	56	0.70	0.45	1.10	0.12	0.71	0.46	1.12	0.14	0.96	0.60	1.53	0.86	0.95	0.60	1.53	0.84
High	51	2	0.33	0.08	1.39	0.13	0.35	0.08	1.46	0.15	0.65	0.15	2.77	0.56	0.63	0.15	2.69	0.53
		***P***_**trend**_	0.046				0.058				0.68				0.65			
Tumor pSTAT3^Y705^ status	***n***	***n***	**HR 5-year**				**HR 5-year**				**HR 5-year**				**HR 5-year**			
Negative	210	21	Ref				Ref				Ref				Ref			
Intermediate	606	28	0.45	0.26	0.79	0.006	0.48	0.27	0.85	0.011	0.66	0.36	1.19	0.17	0.64	0.35	1.17	0.15
High	51	0	No event				No event				No event				No event			
		***P***_**trend**_	0.0008				0.0018				0.060				0.051			
**DEATH**																		
							**Model 1**				**Model 2**				**Model 3**			
	**Total**	**Events**	**Crude**				**Adjusted**				**Adjusted**				**Adjusted**			
Tumor pSTAT3^Y705^ status	***n***	***n***	**HR**	**(95% CI)**		***P*****-value**	**HR**	**(95% CI)**		***P*****-value**	**HR**	**(95% CI)**		***P*****-value**	**HR**	**(95% CI)**		***P*****-value**
Negative	210	36	Ref				Ref				Ref				Ref			
Intermediate	606	72	0.78	0.52	1.17	0.23	0.79	0.52	1.18	0.25	0.98	0.63	1.50	0.91	0.96	0.62	1.48	0.86
High	51	5	0.74	0.29	1.90	0.53	0.76	0.29	1.94	0.56	1.20	0.46	3.14	0.71	1.19	0.45	3.10	0.73
		***P***_**trend**_	0.25				0.27				0.91				0.96			
Tumor pSTAT3^Y705^ status	***n***	***n***	**HR 5-year**				**HR 5-year**				**HR 5-year**				**HR 5-year**			
Negative	210	25	Ref				Ref				Ref				Ref			
Intermediate	606	39	0.54	0.33	0.90	0.018	0.56	0.34	0.93	0.024	0.68	0.40	1.16	0.15	0.65	0.38	1.11	0.11
High	51	1	0.17	0.02	1.24	0.080	0.18	0.02	1.31	0.089	0.29	0.04	2.14	0.22	0.28	0.04	2.11	0.22
		***P***_**trend**_	0.004				0.007				0.078				0.058			

*Model 1: Time between surgery and staining*.

*Model 2: Adjusted for model 1 + age ≥50 years, tumor size, node status, grade III, ER status, HER2 status, and BMI ≥25 kg/m^2^. Missing data for 53 patients for at least one variable*.

*Model 3: Adjusted for model 1 + 2 + adjuvant treatments. Missing data for 55 patients for at least one variable*.

### Effect Modifications Between Patient Characteristics, Tumor Characteristics, Treatment, and Activated STAT3 Levels on Prognosis

Formal interaction analyses were performed to investigate whether there were any effect modifications by age, body constitution, tumor characteristics, or treatments on the association between pSTAT3^Y705^ levels and prognosis. There were no significant effect modifications by age, body constitution, tumor characteristics, chemotherapy, radiotherapy, or trastuzumab. For endocrine treatment, interaction analyses were performed on the 762 patients with ER^+^ tumors. There were no significant interactions between pSTAT3^Y705^ levels and either tamoxifen or AIs on prognosis.

**Figure 3:**
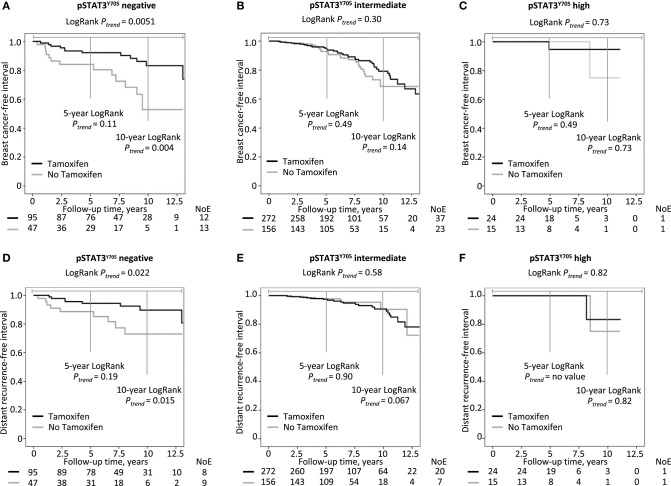
Kaplan-Meier estimates of chemonaïve patients with ER^+^ breast cancers stratified by tamoxifen treatment in relation to pSTAT3^Y705^ level: **(A–C)** breast cancer-free interval, **(D–F)** distant metastasis-free interval.

Chemotherapy-treated patients (*n* = 151) were then excluded since chemotherapy may impact pSTAT3 levels. In the remaining patients, there were significant interactions between pSTAT3^Y705^ negativity and tamoxifen treatment on recurrence-risk (HR 0.38; 95% CI 0.15–0.98; *P*_interaction_ = 0.046) and distant metastasis risk (HR 0.21; 95% CI 0.06–0.77; *P*_interaction_ = 0.019) adjusted for time between surgery and staining. Among the 142 patients with pSTAT3^Y705^-negative tumors, tamoxifen was associated with lower risk of recurrence (HR 0.25; 95% CI 0.09–0.67; *P* = 0.006) and distant metastasis (HR 0.19; 95% CI 0.05–0.74; *P* = 0.017) adjusted for time between surgery and staining, age, BMI, tumor characteristics, and treatments compared with no tamoxifen (see Figures 3A–F). Due to collinearity, we were not able to include HER2 data and trastuzumab in this analysis. In contrast, in patients with intermediate or high pSTAT3^Y705^ levels, there was no difference in recurrence risk or distant metastasis risk between tamoxifen-treated and non-tamoxifen-treated patients.

### Sensitivity Analysis

In the case of bilateral tumors (*n* = 18), pSTAT3^Y705^ from both tumors was available for eight tumors, and the level of pSTAT3^Y705^ differed in three of these tumors. Four patients with bilateral tumors had a recurrence, and three of these had tumor tissue that was evaluable for pSTAT3^Y705^ from both sides. For two patients, the pSTAT3^Y705^ levels differed. Therefore, sensitivity analyses were performed using the lowest pSTAT3^Y705^ value. In general, the associations between pSTAT3^Y705^ and prognosis became slightly stronger, and in the fully adjusted multivariable models, the trends for 5-year distant metastasis-free intervals and 5-year overall survival became significant (both *Ps* ≤ 0.039). The interaction between pSTAT3^Y705^ level and tamoxifen became somewhat weaker for recurrence risk (*P* = 0.071) but remained significant for risk for distant metastases.

## Discussion

The main findings were that the highest pSTAT3^Y705^ levels in primary breast cancer were associated with smaller body sizes and less aggressive tumor characteristics. Patients with pSTAT3^Y705^-negative tumors had an increased risk for early events within the first 5 years, whereas patients with intermediate levels presented with a constant rate of events, and these curves crossed after about 10 years of follow-up. In contrast, patients with the highest pSTAT3^Y705^ levels had a very low recurrence risk across all years. Other studies using different cut-offs for pSTAT3 categories have also reported that higher pSTAT3 levels were associated with a better breast cancer prognosis (10, 11, 13, 30, 33). In the present study, pSTAT3^Y705^ levels were mainly associated with distant metastasis rather than other types of recurrences.

To our knowledge, this is the first study to investigate pSTAT3^Y705^ in relation to body constitution and show that higher tumor-specific pSTAT3 levels are associated with lower BMI, WHR, and smaller breast volumes, which are all good prognosticators for breast cancer (26). The inverse association between pSTAT3^Y705^ levels and body size was unexpected since overweight is associated with chronic low-grade inflammation, and adipose tissue generates large amounts of circulating IL-6 (34) known to activate STAT3 (35). Furthermore, pSTAT3^Y705^ was also associated with other lifestyle factors in the present study, such as alcohol consumption and age at first childbirth. Interestingly, several of the characteristics associated with higher pSTAT3^Y705^ levels are associated with socioeconomic status (36–38), which in turn may influence breast cancer prognosis (39). In spite of caffeic acid being a natural inhibitor of STAT3, there was no association between coffee intake and pSTAT3^Y705^ levels, even when different cut-offs for coffee intake were explored (data not shown).

Similar to the association between body size and pSTAT3^Y705^ levels, increasing age has also been associated with higher systemic IL-6 and pSTAT3 levels (40). In spite of this, higher age was associated with lower pSTAT3^Y705^ levels in the present study. Others have shown positive association (10) or no association between age and pSTAT3^Y705^ levels in breast cancer tumors (13).

In the present study, high pSTAT3^Y705^ levels were associated with favorable tumor characteristics (10). Previously, high levels of pSTAT3^Y705^ have been associated with smaller tumor size, lower histological grade, and lobular type cancer (10, 13, 33). In contrast to our findings, others reported no association between pSTAT3^Y705^ and lymph node involvement (10). In the present study, there was no significant association between ER status and pSTAT3^Y705^, which is in line with some studies (5, 41) but contradicts other studies (10). Although few studies have reported on PR^+^ status, our positive association confirms previous results (5, 10, 13).

Regarding the negative association between HER2 and pSTAT3^Y705^ levels, previous results have been conflicting (10, 13, 33, 42). In contrast to cell models (14) or gene expression studies (6, 43), there was no association between pSTAT3^Y705^ levels and triple-negative breast cancer in the present study. Different cut-offs for ER^+^ and PR^+^ between institutions may partly explain these differences. In Sweden, >10% positively stained nuclei are still used as a cut-off for receptor positivity.

Interestingly, patients with tumors with intermediate pSTAT3^Y705^ suffered a constant rate of recurrences including late recurrences, in contrast to patients with tumors with high pSTAT3^Y705^, who had very few recurrences. Furthermore, patients with pSTAT3^Y705^-negative tumors suffered early recurrences. This picture resembles that seen with ER, where patients with ER^−^ tumors suffer early recurrences, while those with ER^+^ tumors present with a slow but steady rate of recurrences over time (44).

The negative association between pSTAT3 and recurrence risk was further strengthened in the sensitivity analysis, where the bilateral tumor with the lowest pSTAT3^Y705^ levels was used instead of the tumor with the highest pSTAT3^Y705^ level. Dysregulation of the leukemia inhibitory factor receptor (LIFR)-STAT3-SOCS3 signaling pathway leading to pSTAT3 negativity has been associated with metastatic potential in breast cancer (42). Since there is a lack of markers for late breast cancer recurrences, intermediate pSTAT3^Y705^ would merit further study with respect to this outcome.

It is possible that the pSTAT3^Y705^ isoforms, primarily alpha and beta, differ between tumors with intermediate and high levels. These isoforms have different properties, and depending on the distribution of the two isoforms, pSTAT3 may either be tumor promoting or inhibiting (45, 46). The interplay of pSTAT3 with other STATs (11, 13, 47), their negative feedback loops (1, 48), and the tumor microenvironment (43) need to be assessed further. Since the antibody used in the present study detects both the alpha and beta isoforms of pSTAT3^Y705^, we cannot investigate whether the differences in recurrence risk or other prognostic factors between the groups with intermediate and high pSTAT3^Y705^ levels were isoform dependent. It would also have been interesting to evaluate several downstream targets of pSTAT3 to further elucidate whether pSTAT3 in the primary setting mainly activated tumor-inhibiting pathways but that lies outside the scope of the current study (5, 6).

To our knowledge, our study is the first to report a correlation of time between surgery and staining with pSTAT3^Y705^ levels in breast cancer, although a correlation has been reported for other phosphorylated markers (31). Only one previous study assessed time between surgery and staining during pSTAT3 evaluation. In contrast to the present study, where samples with the longest time between surgery and staining were less likely to have high pSTAT3^Y705^ levels, Dolled-Filhart et al. found no time-dependent difference when performing a visual comparison (30).

The frequency of pSTAT3 ^Y705^-positive tumors differs between studies and is dependent on the scoring system and cut-offs. So far, there is no consensus on how to score immunohistochemical staining patterns for pSTAT3. We used three different categories, which is in line with some studies (13, 19), although others used median levels of pSTAT3^Y705^ as a cut-off (10). Rather than using the median, a cut-off for negativity was used in the present study. This cut-off appeared to be biologically relevant with a higher frequency of early compared with later recurrences.

With respect to evaluating pSTAT3^Y705^ levels with TMAs, others have shown that this method yields comparable results to whole sections (10). The tumors evaluable with TMAs were somewhat more aggressive than non-evaluable tumors in the cohort, which may have led to a lower number of tumors with high pSTAT3^Y705^ compared to the entire cohort. The breast cancer cohort is representative of patients treated in Lund (49), and all ages were included. Skåne University Hospital in Lund has a catchment area of approximately 300,000 patients, and no patients were referred to private clinics for breast cancer surgery.

The levels of pSTAT3^Y705^ were evaluated in primary tumors. However, several types of adjuvant breast cancer treatments may impact the pSTAT3 levels (18, 50). When investigating the effect modifications between adjuvant treatment and pSTAT3^Y705^ levels on prognosis, we did not find any with respect to chemo- or radiation therapy. However, for endocrine treatment, there was a significant effect modification between tamoxifen and pSTAT3^Y705^ in chemonaïve patients with ER^+^ tumors. Patients with pSTAT3^Y705^-negative tumors had an improved outcome when treated with tamoxifen compared to AI only or no endocrine therapy. No such difference was seen in patients with pSTAT3^Y705^-positive tumors, indicating that the absence of STAT3 pathway activation may confer primary AI resistance in chemonaïve patients.

We have previously shown that several factors associated with pSTAT3^Y705^ levels, such as high BMI and low alcohol intake, are associated with adherence to endocrine therapy in this cohort (51). However, there was no difference in duration of either tamoxifen or AI with regard to the pSTAT3^Y705^ levels that could explain the effect modification. One group performed mechanistic studies that showed that estradiol could inhibit IL-6 inhibition of STAT3 activation and that this could be reversed by tamoxifen. They demonstrated direct physical interactions between STAT3 and ER that represent a form of crosstalk between STAT3 and ER. This might be one explanation for the effect modification between tamoxifen and pSTAT3 negativity if the negativity was driven by IL-6 (35). Currently, treatments targeting the STAT3 pathway are being evaluated in clinical trials (NCT03195699, NCT02876302). We hypothesize that patients with intermediate pSTAT3^Y705^ levels may be the best candidates in trials with drugs targeting the STAT3 pathway since there were few recurrences in the group with the highest levels.

In conclusion, this study has shown that high levels of tumor-specific pSTAT3^Y705^ were associated with less aggressive tumors and favorable patient characteristics, such as small body size and certain lifestyle factors. High levels of pSTAT3 were associated with a very good prognosis, whereas patients with pSTAT3-negative tumors suffered early recurrences if they had not received tamoxifen treatment as endocrine therapy. In the future, these results may help guide the selection of patients for trials with drugs targeting the STAT3 pathway.

## Data Availability Statement

Datasets for this study will not be made publicly available due to privacy laws.

## Ethics Statement

The studies involving human participants were reviewed and approved by Ethics Committee of Lund University. The patients/participants provided their written informed consent to participate in this study.

## Author Contributions

LN, ES, and HJ: study design, manuscript preparation, statistical analysis, data analysis, and interpretation. HJ, KI, and SB: study supervision. LN, ES, SK, BN, KJ, and HJ: data collection. LN, ES, SK, HT, BN, KJ, SB, and KI: contributed to manuscript review, critical revision for important intellectual content, and read and approved the final draft for submission. All authors are responsible for the manuscript content.

## Conflict of Interest

The authors declare that the research was conducted in the absence of any commercial or financial relationships that could be construed as a potential conflict of interest.

## Abbreviations

AI: aromatase inhibitor
AUDIT: alcohol use disorders identification test
BMI: Body mass index
CI: confidence interval
ER: estrogen receptor
EGF: epidermal growth factor
HR: hazard ratio
HER2: human epidermal growth factor receptor 2
H-score: histoscore
IL-6: interleukin-6
JAK: janus kinase
LIFR: leukemia inhibitory factor receptor
MHT: menopausal hormone therapy
MMP: matrix metalloproteinase
STAT3: signal transducer and activator of transcription 3
OC: oral contraceptives
PIAS3: protein inhibitor of activated STAT3
PR: progesterone receptor
PD-L1: programmed death-ligand 1
pSTAT3: phosphorylated signal transducer and activator of transcription 3
REMARK: reporting recommendations for tumor marker prognostic study
SOCS3: suppression of cytokine signaling 3
TMA: tissue micro array
VEGF: vascular endothelial growth factor
WHO: world health organization
WHR: Waist-to-hip ratio.
